# A Comparison of the Effects of Vitamin B12 and Folic Acid on Gait Recovery and Myelination After Femoral Nerve Injury in Rats

**DOI:** 10.3390/ijms27083664

**Published:** 2026-04-20

**Authors:** Miloš Basailović, Igor Jakovčevski, Milan Aksić, Joko Poleksić, Gorana Basailović, Nevena Divac

**Affiliations:** 1 Faculty of Medicine, Department of Pharmacology, Clinical Pharmacology and Toxicology, University of Belgrade, 11000 Belgrade, Serbia; milos.basailovic@med.bg.ac.rs; 2 Institut für Anatomie und Klinische Morphologie, Universität Witten/Herdecke, 58455 Witten, Germany; 3 Department of Neuroanatomy and Molecular Brain Research, Ruhr University Bochum, 44780 Bochum, Germany; 4 Faculty of Medicine, Institute of Anatomy “Niko Miljanić”, University of Belgrade, 11000 Belgrade, Serbia; milan.aksic@med.bg.ac.rs (M.A.); joko.poleksic@med.bg.ac.rs (J.P.); gorana.basailovic@med.bg.ac.rs (G.B.)

**Keywords:** femoral nerve injury, folic acid, motor recovery, myelination, regeneration, vitamin B12

## Abstract

Peripheral nerve injuries often lead to incomplete recovery despite surgical repair. Vitamin B12 and folic acid have been implicated in nerve regeneration, but their comparative effects have not been systematically evaluated. Twenty-four male Wistar rats underwent femoral nerve transection and were assigned to three groups: control, vitamin B12 (2500 µg/kg weekly, subcutaneous), and folic acid (40 mg/L in drinking water). Functional recovery was assessed over eight weeks using foot-base angle (FBA) during beam walking. Histological analysis evaluated axon counts and myelination (g-ratio). Both treatments accelerated early gait recovery compared to controls, with significant FBA improvement at week 4 (*p* < 0.05). Vitamin B12 produced sustained functional benefits through week 8 and superior myelination (lower g-ratio, *p* < 0.0001), whereas folic acid increased axon numbers but did not enhance myelin thickness or late-phase recovery. High-dose vitamin B12 significantly improves structural and functional outcomes after femoral nerve injury, while folic acid primarily supports early axonal regrowth. Vitamin B12 represents a promising pharmacological adjunct for peripheral nerve repair. Further research should explore optimal dosing strategies and long-term effects in clinical settings. To our knowledge, no prior study has directly compared the effects of folic acid and vitamin B12 supplementation within the rat femoral-nerve model, providing the rationale for the present head-to-head design.

## 1. Introduction

Peripheral nerve injury (PNI) is a condition that may affect one or more nerves, involving varying degrees of structural damage to both neural and surrounding connective tissues, and is typically accompanied by partial or complete loss of function distal to the lesion site. Peripheral nervous system tissue exhibits significantly greater regenerative capacity than the central nervous system [1]. Following axonal injury, peripheral neurons activate a cascade of signaling pathways involving growth-associated genes, leading to the reformation of a functional growth cone and axonal regeneration. Degenerative processes are also involved in nerve repair, serving to remove debris and molecules detrimental to regeneration [2]. These degenerative processes occur both proximally and distally to the injury site but differ significantly [3]. Wallerian degeneration (WD) is an orchestrated anterograde breakdown of the distal axon segment after injury, with a rapid activation phase and well-defined stages in the peripheral nervous system (PNS). In rats, WD begins within hours and is morphologically detectable within 1–3 days, and transcriptomic profiling delineates three phases: 0–6 h (acute response), 6–24 h (preformation), and 4 days–4 weeks (execution of WD) [4]. In vivo MR studies demonstrate resolution of active WD signals at ~30 days after crush injury and at 45–54 days after transection (neurotomy) [5]. Axonal regeneration (sprouting) from the proximal stump typically starts after a short latency of ~4 days [6]. The proximal stump experiences a short segment of retrograde (traumatic) degeneration that typically extends to the first node of Ranvier (i.e., approximately one internode). This proximal change precedes regrowth from the cut end [6]. The fastest motor axons in rats elongate at ~3.2 mm/day [6], measured after sutured transection in the sciatic nerve model, whereas clinical practice often adopts a reference rate of ~1 mm/day for human peripheral nerves [7], with neurographic data indicating that human WD starts no later than day 4 and reaches its amplitude plateau around day 8 [8]. Functional recovery requires that the regenerating axon reach its target organ (muscle) through Schwann cell-formed endoneurial pathways (bands of Büngner), which simultaneously clear myelin and provide trophic support [9]. Time to reinnervation is therefore proportional to distance. Despite the significant regenerative potential of peripheral nerves, advanced surgical techniques, and physical therapy, complete functional recovery is often not achieved following severe injuries. Numerous experimental and clinical studies have sought to identify pharmacologically active substances that could enhance peripheral nerve regeneration. However, no pharmacological agents have yet been approved for clinical use in the treatment of nerve injuries [10,11]. Several experimental approaches have demonstrated improved nerve regeneration and functional recovery in animal models, but translation into clinical practice remains unrealized [12]. Among the substances most commonly used in the treatment of peripheral nerve injuries and disorders are B-complex vitamins. Vitamin B12 (cobalamin) has proven to be the most effective among the B vitamins in promoting peripheral nerve regeneration following injury. This vitamin serves as a crucial coenzyme in numerous biochemical processes essential for normal nervous system function. Several forms of vitamin B12 exist, with methylcobalamin and deoxyadenosylcobalamin being the active forms. A deficiency in vitamin B12 leads to a lack of methionine, which is necessary for the synthesis of membrane phospholipids and myelin [11]. Myelopathy and neuropathy are the most prominent clinical manifestations of vitamin B12 deficiency [13]. Vitamin B12 facilitates axonal growth, increases the axonal diameter, promotes Schwann cell proliferation during peripheral nerve regeneration, enhances axonal myelination, and upregulates the expression of certain neurotrophic factors, particularly brain-derived neurotrophic factor (BDNF) [14]. In combination with vitamins B1 and B6, it has shown beneficial clinical effects in neurodegenerative diseases and is routinely used in clinical practice. In addition to vitamin B12, folic acid plays a vital role in the development and regeneration of the nervous system [15,16]. It is well established that folic acid supplementation can prevent neural tube defects [17,18]. Folic acid, in combination with uridine monophosphate and vitamin B12, has demonstrated therapeutic effects in carpal tunnel syndrome, a chronic nerve injury [19]. In a rat model of acute sciatic nerve injury, folic acid was shown to enhance regeneration and promote myelination [20]. Furthermore, studies have shown that folic acid increases nerve growth factor (NGF) gene expression and stimulates NGF secretion by Schwann cells [21,22]. In our study, we aimed to compare the effects of supplementation with vitamin B12 and folic acid on gait recovery and nerve regeneration, following femoral nerve transection.

## 2. Results

### 2.1. Gait Recovery After Femoral Nerve Injury

**Figure 1:**
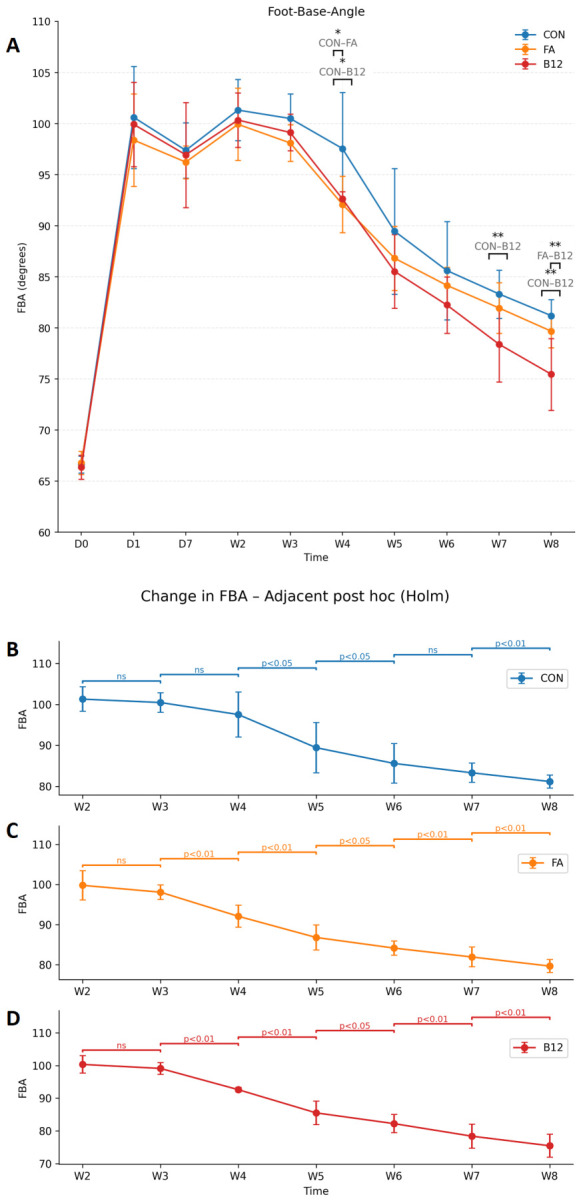
Time course of recovery of the foot-base angle (FBA); between-group comparisons at matched time points (**A**). Shown are mean values ± SD in rats treated with vitamin B12 (B12) and folic acid (FA) and in control rats without treatment (CON). Brackets denote pairwise comparisons at the same timepoints; asterisks indicate significance level (* *p* < 0.05; ** *p* < 0.01, Tukey’s Honestly Significant Difference (HSD), n = 8 rats per group). (**B**–**D**) show within-group adjacent-time comparisons, starting from W2, highlighting significant week-to-week changes for each group. Shown are mean values ± SD in rats treated with vitamin B12 (B12) (**D**), folic acid (FA) (**C**), and in untreated control rats (CON) (**B**). *p*-values indicate differences between adjacent time points within each group (*p* < 0.05 or *p* < 0.01, repeated-measures ANOVA with Holm-adjusted paired *t*-tests; n = 8 rats per group).

Single-frame video analysis has shown that rats after unilateral femoral nerve injury lose function of the quadriceps muscle, shown by the loss of their knee-extensor function and of the hip-flexor component of the rectus femoris, with a subsequent increase in the foot-base angle from approximately 65 to 100 degrees (Figure 1A). A two-way repeated-measures ANOVA was conducted with group (CON, FA, B12) as the between-subjects factor and time (W2–W8) as the within-subjects factor. We selected W2 as the initial time point for analyzing the course of nerve recovery and regeneration because on the day after transection (D1) the process of retrograde degeneration is still ongoing [4], and there is a latency of approximately four days before sprouting begins from the proximal stump [6]. Considering that in this experimental model transection was made 3 mm proximal to the bifurcation with a 3 mm gap, plus the segment of proximal degeneration was up to the first node of Ranvier [1,3], the axon must bridge approximately 6–7 mm before reaching the motor branch and thereafter must bridge the length of the branch itself before arriving at the neuromuscular junctions (NMJs) in the quadriceps. At a regeneration rate of 3.2 mm/day and with a latency of ~4 days, the earliest contact with the neuromuscular junction is expected to be approximately 9–13 days after injury [4,8]. At time point D0, the healthy, pre-injury state of the nerve was assessed; therefore, it is not meaningful to include D0 together with the time points used to monitor recovery. At time point D7, residual postoperative pain may still be present, which we presume caused hip adduction (keeping the thigh close to the body) and consequently a reduction in FBA due to compensatory decreased weight-bearing on the injured side (Figure 1A). In view of all these considerations, it is methodologically fully justified to use W2 as the starting time point for statistical analysis using a two-way ANOVA for repeated measures with Tukey post hoc testing. The analysis revealed a significant main effect of time (*p* = 0.00000; *p* < 0.01), indicating that FBA scores changed systematically throughout the recovery period across all groups. A significant main effect of group was also observed (*p* = 0.00000; *p* < 0.01), suggesting overall differences in FBA levels between treatment conditions. Critically, the analysis demonstrated a significant Group × Time interaction, indicating that the pattern of FBA recovery over time differed across the three groups (*p* = 0.03497; *p* < 0.05). This means that recovery profiles differed across groups: the rate and extent of FBA recovery depended on the treatment administered. To further explore where these differences occurred, Tukey HSD post hoc comparisons were performed at each time point. These analyses showed no significant differences between groups at the beginning of the study (D0); none of the groups exhibited an initial advantage, as all *p*-values were well above 0.05. There were also no significant differences between groups at time points D1–W3 (*p* > 0.05). Some degree of gait recovery followed over the next 8 weeks in all groups of animals, but B12-treated rats recovered better starting at 4 weeks after injury, which remained throughout the rest of the recovery period (Figure 1A). Rats treated with folic acid had better recovery than control rats also at 4 weeks post-injury, but during the second month after injury, they did not statistically differ from control animals (Figure 1A). A statistically significant difference in FBA values was observed between the B12 group and the control group (*p* = 0.03; *p* < 0.05), as well as between the folic acid group and the control group (*p* = 0.0156; *p* < 0.05) at 4 weeks post-injury (W4). No significant difference was found between the B12 and folic acid group at this time point. Furthermore, at 7 weeks post-injury (W7), a statistically significant difference in FBA was detected between the B12 and control group (*p* = 0.0074; *p* < 0.01), whereas the folic acid group did not differ significantly from the control. Finally, at 8 weeks post-injury (W8), FBA values differed significantly between the B12 and control group (*p* = 0.0003; *p* < 0.01), as well as between the B12 and folic acid groups (*p* = 0.006; *p* < 0.01), while no significant difference was observed between the folic acid and control groups (Figure 1A).

Regarding recovery progress (FBA reduction) within groups (Figure 1B–D), both the B12 and folic acid groups exhibited statistically significant differences in FBA between consecutive time points starting from W3–W4, with a consistent trend of significant weekly reduction continuing until the end of the observation period. In the control group, a statistically significant decrease in FBA was observed only after 5 weeks, beginning with the W4–W5 interval, and this trend persisted through the remainder of the study, except for the W6–W7 interval.

### 2.2. Numbers of Axons and the Degree of Myelination in the Motor Branch

**Figure 2:**
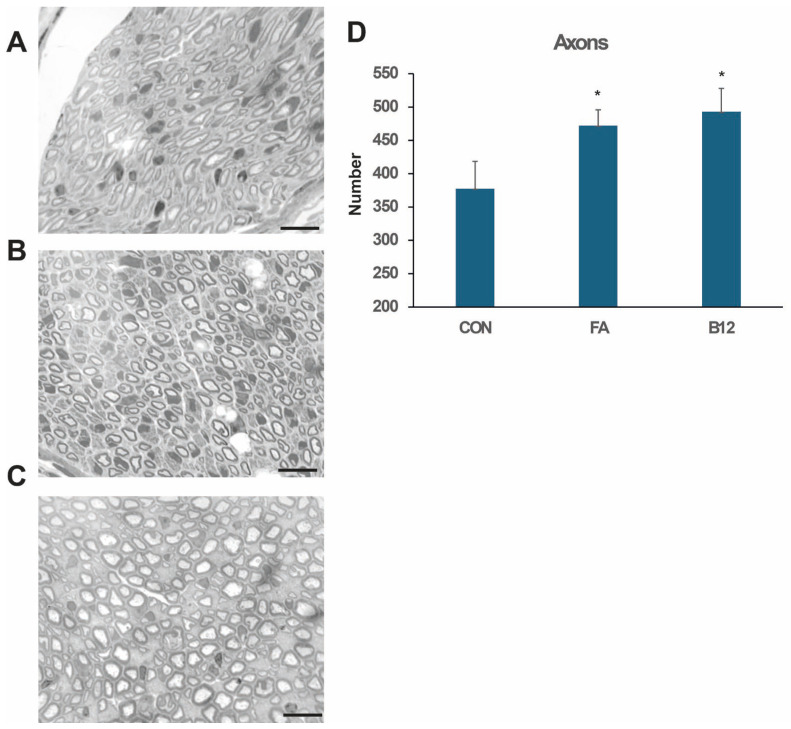
The number of myelinated nerve fibers in the motor branch of the regenerated femoral nerve after eight weeks of recovery following injury. Representative images show myelinated axons in the motor branch of control (CON; (**A**)), folic-acid-treated (FA; (**B**)) and vitamin-B12-treated (B12; (**C**)) rats. Scale bars: 25 μm. The number of myelinated axons in the motor (quadriceps) branch of control rats (CON), as well as rats treated with folic acid (FA) and vitamin B12 (B12), 2 months after injury (**D**). Data are shown as the mean + SD. Asterisks indicate difference from the control group (one-way ANOVA with Tukey’s post hoc test). n = 6 nerves/group.

Numbers of axons and the degree of myelination in non-injured and regenerated nerves were estimated in toluidine blue-stained semithin sections. The total numbers of myelinated axons in the motor branch of the injured femoral nerve 2 months after injury were higher in both folic-acid- and vitamin B12-treated rats than in controls (Figure 2D).

**Figure 3:**
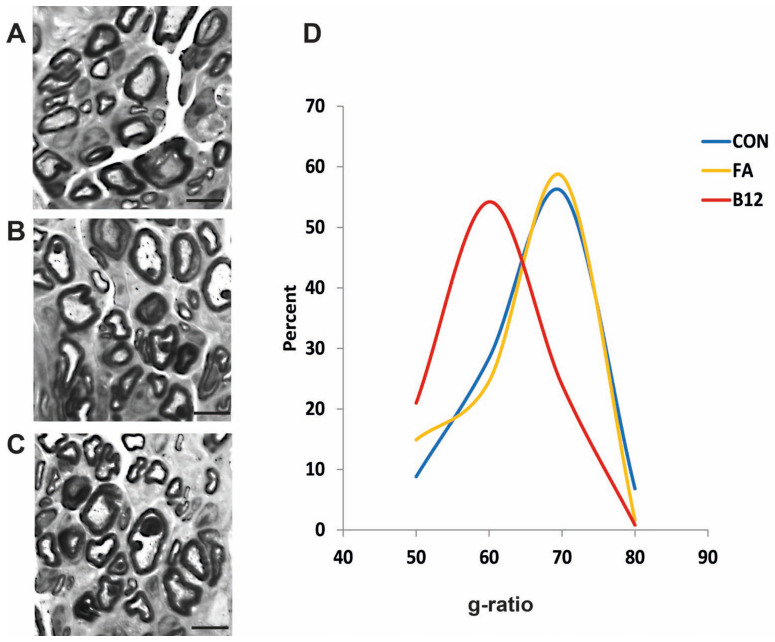
Myelinated nerve fibers in motor nerve branches. Representative images show myelin on axons in the motor branch of control (CON; (**A**)), folic-acid-treated (FA; (**B**)) and vitamin-B12-treated (B12; (**C**)) rats. Scale bars: 5 μm. (**D**) Normalized frequency distributions for g-ratios (axon/fiber diameters) of the injured femoral nerve in CON and FA- and B12-treated rats, 2 months after injury. Per nerve, 75 axons were analyzed, from 6 nerves per treatment. The distribution in B12 nerves is different from the distribution of the CON and FA groups (*p* < 0.0001, Kolmogorov–Smirnov test).

Analysis of myelination, measured by the g-ratio, revealed differences between the control and folic-acid-treated group from the vitamin-B12-treated group in regenerated nerves, indicating better myelination in B12 treated rats, in comparison with control treated and folic-acid-treated animals (Figure 3A–D). There was no statistically significant difference between folic-acid-treated animals and controls (Figure 3D).

### 2.3. Terminal Serum Vitamin Levels (Proof of Exposure)

**Figure 4:**
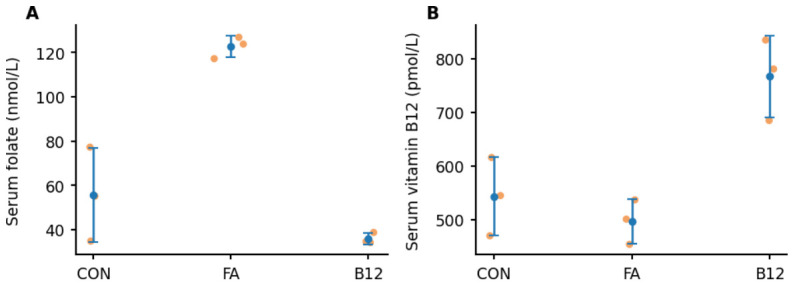
Terminal serum folate and vitamin B12 levels (proof of exposure). (**A**) Serum folate (nmol/L) and (**B**) serum vitamin B12 (pmol/L) measured in a random subset of animals (n = 3 per group) at euthanasia, 7 days after the last B12 injection. Orange dots indicate individual animals; blue circles with error bars indicate mean ± SD. Measurements were performed to confirm systemic exposure and were not intended to establish pharmacokinetic equivalence across regimens.

Terminal serum measurements were performed to confirm systemic exposure to the administered vitamins. Blood was collected at euthanasia (7 days after the last B12 injection), and a random subset of three serum samples per group was analyzed for folate (nmol/L) and vitamin B12 (pmol/L). Serum folate concentrations were higher in the FA group (122.58 ± 4.92 nmol/L; range 117.18–126.82) than in controls (55.74 ± 21.19 nmol/L; range 34.86–77.23) and the B12 group (35.88 ± 2.51 nmol/L; range 34.19–38.76). Conversely, serum vitamin B12 concentrations were higher in the B12 group (767.00 ± 75.97 pmol/L; range 685–835) than in controls (543.67 ± 73.01 pmol/L; range 470–616) and the FA group (497.33 ± 41.62 pmol/L; range 454–537). These terminal measurements served as proof of exposure and were not intended to establish pharmacokinetic equivalence across regimens (Figure 4).

## 3. Discussion

This study investigates the effects of high doses of vitamin B12 and folic acid on femoral nerve regeneration and functional recovery following transection with entubulation of the proximal and distal stumps, leaving a 3 mm gap between them. Our findings indicate that high-dose vitamin B12 significantly enhances both functional and structural recovery of the femoral nerve in rats eight weeks post-injury. Functional recovery, assessed by the foot-base angle, was superior in the B12 group compared to both the folic acid and control groups. Furthermore, myelination quality, reflected by the g-ratio, was markedly better in the B12 group than in the FA group and control, with no significant difference between FA and control. The number of myelinated axons in the motor branch of the injured nerve was higher in animals treated with vitamin B12 and folic acid compared to the control. Although the axon count was greater in B12 than FA, this difference did not reach statistical significance.

To monitor and quantify functional recovery, we employed the single-frame motion analysis method, using FBA as the parameter of interest. SFMA enables detection of subtle differences in FBA values, allowing precise, reliable, and objective assessment of gait recovery in this experimental model. SFMA is a validated method with strong intra-observer and inter-observer agreement for repeated measurements, ensuring accuracy and reproducibility [23]. Based on FBA values over time, we conclude that high doses of vitamin B12 and folic acid accelerate early-phase recovery. This is evident from the significant reduction in FBA and improvement in gait after four weeks (W4) in treated groups compared to control. Additionally, analysis of weekly FBA trends within groups reveals that the initial improvement in motor function occurs between W3 and W4 in B12 and FA groups, whereas in the control it is delayed until W4–W5. This early improvement may be attributed to the greater number of myelinated axons in B12 and FA groups, potentially enabling reinnervation of more motor units during the early recovery phase. After W4, significant intergroup differences in FBA diminish by W5, likely due to the increase in reinnervated motor units in the control group and the associated reduction in FBA, although treated groups still exhibit slightly better values. Beyond this point, no significant difference persists between FA and the control, despite FA maintaining marginally better values, whereas B12 continues to show superior recovery compared to both FA and the control. At W7, FBA is significantly better in B12 than in controls, and at W8, it is better in B12 than both in FA and the control (Figure 1A). This sustained improvement in gait recovery in B12-treated animals is consistent with superior axonal myelination compared to other groups. Short follow-ups (≤4–6 weeks) are common in vitamin-based PNI studies and primary index early recovery; by extending to 8 weeks, our design differentiates transient early gains from sustained remyelination-linked benefits and prevents a false early equivalence between FA and B12. Given differences in routes and dosing regimens, the comparison is not strictly like-for-like; nonetheless, the related literature allows us to position our outcomes relative to comparable models.

The reason why functional recovery in the control group approaches that of FA, despite FA having a significantly higher axon count, likely lies in the remarkable sprouting capacity of regenerating axons. Specifically, collateral sprouting enables individual motoneurons to reinnervate additional muscle fibers. Following injury, axons can reinnervate up to five times more muscle fibers than healthy motoneurons initially innervate [24]. Thus, preservation and recovery of approximately 20% of motoneurons may suffice for complete reinnervation and restoration of muscle control. Another factor contributing to the generally good functional recovery in controls may be the phenomenon of preferential motor reinnervation (PMR) [25,26,27,28]. In rats, PMR means that a greater proportion of motoneuron axons regenerate into the muscle branch rather than the sensory branch after femoral nerve transection proximal to bifurcation [26,27,28], despite equal opportunity for regrowth into either branch. In contrast, injury to nerves innervating multiple muscles, some of which may act as antagonists (e.g., sciatic or facial nerves), can potentially lead to disruption of myotopic organization due to misdirected regenerating motoneuron axons [29]. This may result in loss of coordination between muscles, significantly impacting motor function tests. At the end of the observation period (8 weeks), residual functional deficits persisted in all groups, and previous studies have shown that such deficits remain even 20 weeks post-injury, even when immediate end-to-end suture repair of transected nerve is done [23].

Regarding structural recovery, our results align with findings from other studies. Sağır et al. reported [30] that folic acid significantly increases the number of myelinated axons following sciatic nerve crush injury, without improving myelin thickness, consistent with our findings. Other studies have likewise reported that folic acid promotes axonal regeneration after peripheral nerve injury by increasing axon number, although myelination quality (g-ratio) was not assessed in those cohorts [20,30,31]. Folic acid has been reported to facilitate peripheral nerve repair possibly by activating the DNM3–AKT signaling axis. Kang et al. (2025) [31] suggested that folic acid enhances peripheral nerve repair by restoring the methionine-cycle balance (normalizing SAM/Hcy), which in turn drives epigenetic demethylation and histone modifications at the DNM3 promoter and upregulates DNM3 expression. Elevated DNM3 has been shown to interact with protein kinase B (AKT), leading to activation of the AKT pathway, which promotes neuronal survival by upregulating anti-apoptotic proteins (BCL-2), downregulating pro-apoptotic factors (Bax, caspase-3), and stimulating autophagy-related markers (LC3, Beclin-1). The authors suggest that folic acid actually normalizes injury-induced dysregulation of BCL-2, Bax, Caspase-3, LC3, and Beclin-1, restoring them toward control levels and thereby reducing apoptosis and fine-tuning autophagy via the DNM3–AKT axis. These changes (BCL-2, Bax, Caspase-3, LC3, and Beclin-1) were demonstrated in vitro in both neurons and Schwann cells [31]. Within the framework of Wallerian degeneration, Schwann cell-mediated autophagy (myelinophagy) contributes to timely myelin-debris clearance and formation of permissive Büngner bands, thereby providing a “clean path” for advancing regenerating axons [4,9]. In this light, the FA-associated normalization of LC3/Beclin-1 reported by Kang et al. [31] can be interpreted as a pro-clearance signal that supports early axonal advance rather than being a direct driver of late-phase myelin thickening. Additionally, AKT activation has also been linked with an increase in antioxidant enzyme activity (SOD, GPx), mitigating oxidative stress [31,32,33]. Beyond its signaling role, DNM3 contributes to cytoskeletal remodeling, axonal growth, and synaptic plasticity, all of which are essential for functional recovery following nerve injury [34,35]. These mechanistic mediators were not assayed in our dataset and are cited here in a hypothesis-generating context. The same caveat applies to the vitamin B12 mechanistic remarks presented further below. In a study examining folic acid’s effects on Schwann cell proliferation, migration, and nerve growth factor secretion, Kang et al. observed significant Schwann cell proliferation [22] both in vitro, in culture, and in vivo, in the nerve, and distal to the injury, in folic-acid-treated groups compared to controls [22]. They also reported that folic acid increases NGF secretion from Schwann cells, and because NGF directly promotes axonal outgrowth, this supports a plausible paracrine mechanism contributing to FA-mediated nerve repair [22]. Therefore, our findings confirm that folic acid promotes an increase in the number of myelinated axons (and consequently Schwann cells) during regeneration after injury, which is important for early-phase recovery. However, it does not confer beneficial effects on myelin thickness, explaining why gait recovery does not differ significantly from the control in the late phase of recovery. Although most studies investigating the impact of folic acid on nerve regeneration utilize parenteral administration, our study employed the oral route via drinking water at a concentration of 40 mg/L. Based on the average daily water consumption in rats, this concentration corresponds to a dosage of approximately 4–4.8 mg/kg/day, as detailed in the Materials and Methods section. This concentration of 40 mg/L is characterized as a very high oral dose for rats [36]. Considering that the recommended dietary allowance for folic acid in rats is 1.0 mg/kg of diet [37], and given that gastrointestinal absorption is highly efficient with substantial oral bioavailability [38], this approach ensured a consistent, elevated daily influx of folic acid and stable, high plasma levels.

With regard to the impact of vitamin B12 on structural recovery of the femoral nerve following injury, our findings indicate that high doses of vitamin B12 significantly contribute to peripheral nerve regeneration. Similar to folic acid, B12 promoted a greater number of myelinated regenerating axons compared to the control group, but also improved myelination quality and myelin thickness. Previous studies have unequivocally demonstrated that vitamin B12 enhances axonal regeneration after nerve injury [14,39,40,41]. B12 has been proposed to support neuronal survival and neurite outgrowth, potentially through activation of Erk1/2 and Akt signaling pathways via the methylation cycle, as reported by Okada et al. (2010) [40]. They concluded that the potential mechanism of methylcobalamin (MeCbl) involves its action within the methylation cycle as a cofactor for methionine synthase, raising S-adenosylmethionine (SAM) and producing sustained activation of ERK1/2 and Akt in neurons, thereby enhancing neurite outgrowth and neuronal survival. Pharmacologic evidence that a methionine-synthase inhibitor (Nbtd) abolishes MeCbl’s effects, whereas SAM mimics and rescues them, and that MEK or PI3K blockade eliminates the neuritogenic/neuroprotective response, causally links the methylation cycle and ERK/Akt signaling to these phenotypic outcomes. The authors further propose, without direct target demonstration, that SAM-dependent carboxyl-methylation of CAAX proteins such as Ras may act upstream of ERK/Akt (and potentially mTOR), offering a mechanistic basis for signal persistence. Concordant in vivo findings in a sciatic nerve injury model—where continuous high-dose MeCbl accelerates functional recovery and increases myelinated axon counts and MBP/NF-positive areas—support the translational relevance of these molecular effects [40]. It is well established that vitamin B12 plays a critical role in the methionine cycle and is essential for phospholipid and myelin synthesis [42,43]. B12 has been shown to accelerate Schwann cell differentiation [44], increase their number [41], and upregulate myelin basic protein expression [45], thereby promoting remyelination. Furthermore, B12 improves myelin compactness [40,45], meaning that myelin layers are tightly packed without vacuoles or discontinuities, hallmarks of a healthy or well-restored myelin structure. Compact myelin ensures rapid and reliable nerve impulse conduction. Regarding the potential mechanisms of vitamin B12 in Schwann cells, Nishimoto et al. (2015) [44] report that methylcobalamin (MeCbl) transiently suppresses Erk1/2 activity in Schwann cells and, under differentiation conditions, significantly increases the expression of MBP and ATP-citrate lyase (Acly; lipid biosynthesis), while accelerating the formation of MBP-positive myelin segments in DRG–Schwann cell co-cultures. In vivo, in an LPC-induced demyelination model, they confirm remyelination and functional recovery by histologic and electrophysiological/behavioral readouts. The authors conclude that MeCbl directly promotes Schwann cell differentiation/remyelination, likely via a reduction in Erk1/2 signaling—a recognized negative regulator of myelination in SCs—rather than via proliferation; they emphasize that the effect is expressed during the differentiation phase, not during the proliferative phase of Wallerian degeneration. This contrasts with the neuronal effect described by Okada et al., 2010, where MeCbl increases Erk1/2 and Akt activity via the methylation cycle, suggesting that the target cell type may determine the direction of ERK modulation [44]. We reiterate that these mechanistic mediators were not assayed in our dataset and are cited here in a hypothesis-generating context. In addition to increasing the number of regenerated nerve fibers, other reported effects of vitamin B12 on peripheral nerve recovery include greater fiber diameter and increased myelin thickness [14,39,40]. In our study, myelination analysis revealed a significantly higher proportion of well-remyelinated fibers with thick myelin (as measured by the g-ratio) in the B12-treated group compared to both the control and folic acid groups. The greater number of regenerated fibers and thicker myelin observed in the B12 group, relative to folic acid and control groups, align with the functional recovery outcomes following femoral nerve injury in our study. Vitamin B12 demonstrated superior efficacy compared to folic acid during the later stages of gait recovery. While SFMA/FBA captures longitudinal gait recovery in the rat femoral-nerve model and is widely used/validated, we did not include electrophysiology (e.g., CMAP/NCV), ex vivo contractile force, or muscle atrophy measurements in this study. We therefore interpret the lower g-ratio observed with B12 as a structural substrate consistent with the FBA improvement, rather than a direct demonstration of enhanced conduction. In our study, we obtained positive results regarding the impact of vitamin B12 on femoral nerve regeneration. Although other investigators have also employed ultra-high dosages [39], we opted to administer the total weekly dose of 2500 µg/kg of rat body weight as a single subcutaneous injection instead of in five divided doses. This dosing regimen facilitates a high peak concentration, while the highly efficient enterohepatic recirculation of vitamin B12 maintains stable, elevated serum levels between doses [46,47,48,49]. For long-term studies, subcutaneous (SC) administration is considered the safest route as it is minimally invasive, induces the least stress in animals, and minimizes the risk of vital organ damage or hemorrhage—risks inherently associated with intraperitoneal (IP) or intramuscular (IM) routes [50]. The substance often remains at the injection site, diffusing gradually into surrounding tissues before entering the bloodstream (depot effect); in rats, the subcutaneous space is particularly loose, allowing for lateral fluid dispersion, which further modulates systemic entry. Furthermore, subcutaneous administration results in slower absorption compared to IM or IP routes due to lower vascularity, providing a distinct advantage when a more stable blood concentration is required without abrupt fluctuations [51].

Given the differences in routes and dosing regimens, and acknowledging that the comparison is not strictly like-for-like, the related literature allows us to position our outcomes relative to comparable models. Our late-phase myelin advantages with vitamin B12 are concordant with independent studies showing enhanced remyelination and functional recovery with high-dose methylcobalamin under different dosing schedules and delivery modes [40,44], supporting an intrinsic biological effect rather than a schedule-specific peak. By contrast, our folic-acid histology—more myelinated axons without thicker myelin—agrees with studies using daily parenteral FA [20,30]. Mechanistically, FA can promote Schwann cell proliferation/migration and NGF secretion [22], which is consistent with increased axon counts without a proportional enhancement of myelin thickness. To our knowledge, this is the first study to compare folic acid and vitamin B12 within the rat femoral-nerve model, which may explain the absence of directly comparable datasets in prior reports.

**Limitations of the study.** We used an acute femoral-nerve transection model with a silicone conduit and a 3 mm gap (representing the most severe injury grade—transection with a gap), which does not capture other injury types (e.g., compression, ischemia). We studied only male Wistar rats over an 8-week period; longer recovery trajectories and sex differences were not assessed. We tested specific supplementation regimens (vitamin B12 s.c. once weekly; folic acid continuously in drinking water) without alternative routes of administration or other dosing schedules. Serum folate and vitamin B12 were not monitored longitudinally (no pharmacokinetic time-course), but terminal serum levels were measured in a random subset (n = 3/group) as proof of exposure. Therefore, differences in exposure profiles between the subcutaneous B12 regimen and the drinking-water folic-acid regimen cannot be fully excluded and may contribute to effect sizes. We interpret efficacy under the tested regimens and note that our histological pattern is concordant with independent reports using alternative dosing/routes for methylcobalamin (late-phase remyelination benefit) and folic acid (increase in myelinated axon counts without thicker myelin). Functional analysis relied on SFMA/FBA without electrophysiology (e.g., CMAP, conduction velocity) or quantitative muscle strength testing, so translation to nerve conduction and contractility is indirect. Histology was limited to light microscopy of semithin sections and the g-ratio, without molecular markers (e.g., MBP, BDNF, NGF) that could further elucidate remyelination mechanisms.

## 4. Materials and Methods

### 4.1. Animals

The study was conducted on 24 male Wistar rats, weighing between 250 g and 300 g, divided into three groups, with two experimental groups and one control group. The number of animals was determined based on previous publications related to the femoral nerve lesion model. Pre-defined exclusion criteria (e.g., wound dehiscence, foot injury unrelated to the lesion, intra-operative complications, behavioral non-compliance or inconsistent gait patterns during baseline testing preventing reliable kinematic data collection) were set a priori; no animals met these criteria. Following the surgical lesion of the left femoral nerve, the rats were monitored for 8 weeks to evaluate nerve recovery. Animals were kept under standard conditions (room temperature, 12/12 light–darkness cycle, single-caged, with food and water ad libitum). Animals were allocated to control (CON), folic acid (FA), and vitamin B12 (B12) groups (n = 8 per group) using a computer-generated permuted block randomization (1:1:1) with body-weight stratification. This study was approved by a decision No. 323-07-9632/2021-05 issued by the Veterinary Directorate of the Ministry of Agriculture, Forestry and Water Management of the Republic of Serbia.

### 4.2. Surgical Procedures

**Figure 5:**
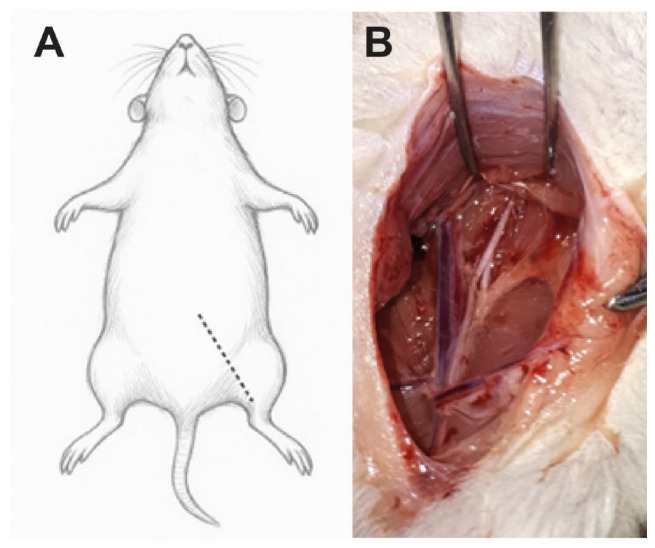
Femoral nerve injury surgery in rats. (**A**) Schematic drawing of a rat with dotted line showing a skin opening section. (**B**) Exposed femoral nerve on the underlying psoas muscle with femoral (mixed, right) and saphenous (sensory, left) branches. Nerve is transected 3 mm before the branching point.

The animals were anesthetized via intraperitoneal injection containing a combination of ketamine–xylazine (ketamine: 100 mg/kg, xylazine: 8 mg/kg). A subcutaneous (s.c.) injection of analgesic (ketoprofen: 5 mg/kg) was administered preoperatively, followed by two additional doses at 24 h intervals. Upon induction of anesthesia, the surgical field was prepared by removing the fur. The animal was then fixed to a surgical table (as shown in Figure 5).

An incision through the skin and subcutaneous fat tissue (indicated by the dashed line in Figure 5A) provided access to the left femoral fossa. Careful dissection of the femoral fossa revealed the nerve and vascular structures (Figure 5B). The femoral nerve was transected 3 mm proximal to its bifurcation. The proximal and distal ends were inserted 1 mm into a silicone tube of 5 mm length, leaving a 3 mm gap filled with physiological saline. The ends of the transected nerve and the tube were sutured with 10-0 thread to maintain the desired position. The skin and subcutaneous fat tissue were returned to their original position, and the skin incision was closed with absorbable 5-0 sutures. Surgeries were performed over several days; within each day, the order of procedures was randomized and balanced across groups to minimize time-related drift. Postoperative analgesia and husbandry were identical across groups according to the protocol.

### 4.3. Monitoring of Locomotor Recovery, Dosage, and Administration of Vitamins

Postoperatively, the animals received weekly s.c. injections starting from the day of surgery, ending with the last injection 7 weeks after surgery (8 doses in total), according to their experimental group. The control group received only the injection vehicle, the B12 group received s.c. injections of vitamin B12 (methylcobalamin, at a dose of 2500 µg/kg), and the folic acid group received the injection vehicle, along with drinking water enriched with folic acid (at a concentration of 40 mg/L). These doses are considered high for rats [36,39]. We opted to administer the entire weekly dose of vitamin B12 at once and to provide folic acid supplementation orally via drinking water as a more convenient approach for the animals, in order to avoid daily injections and minimize the induction of stress during the trial. On each scheduled testing week, SFMA/FBA recording was performed prior to the weekly injection; injections were administered immediately after completion of the behavioral session. Studies in rats show that folic acid exhibits linear pharmacokinetics at doses commonly used for supplementation [52]. This means that a larger single dose (e.g., weekly) proportionally elevates the serum level in the short term (high C_max), but it is also eliminated more rapidly via the kidneys. In rats, the liver serves as the principal reservoir for folate storage, although its storage capacity is far smaller than that for vitamin B12. Research suggests that daily intake maintains more stable hepatic steady-state levels, whereas with large individual doses (which would simulate weekly dosing) a greater fraction of folate may be excreted in urine before tissues can absorb it, especially if the capacity of folate transporters is exceeded [53]. Human studies likewise indicate more effective supplementation with daily compared with weekly dosing [38]. Folic acid has high oral bioavailability, and most individuals—including those with many malabsorption conditions—effectively absorb folic acid orally; therefore, oral administration effectively and reliably increases folate levels without injections [54]. In line with this, continuous oral supplementation is methodologically appropriate, both to avoid daily injections and to achieve stable folic acid levels. Using standard husbandry guidelines for adult Wistar rats of 10–12 mL/100 g/day, a 250–300 g male typically consumes ≈25–36 mL of water per day [55,56,57]. At the studied folic acid concentration of 40 mg/L in drinking water, this corresponds to ≈1.00–1.44 mg/day, i.e., ≈4.0–4.8 mg/kg/day and ≈28.0–33.6 mg/kg/week. We used subcutaneously administered vitamin B12 to bypass gastrointestinal absorption dependent on intrinsic factor and ileal receptors and to ensure robust systemic levels, consistent with clinical and preclinical practice [58]. Both parenteral and oral high-dose B12 regimens can raise vitamin levels and correct deficiency, but parenteral dosing is still often chosen when consistent exposure is required or when modeling parenteral clinical regimens [59]. Maintaining high levels of this vitamin with once-weekly dosing is feasible because of the large hepatic storage capacity for B12 and its enterohepatic recirculation [46,47]. The extent of enterohepatic recirculation of vitamin B12 is highly significant and is considered one of the most efficient mechanisms of nutrient conservation in the body, in both humans and rats [48,49]. Rat studies show that this mechanism is sufficiently active and that serum B12 levels remain stable for weeks even after complete cessation of dietary intake [46,47]. Therefore, the choice of dosing schedule and route of administration for vitamin B12 in this study is methodologically appropriate and ethically justified. Both selected routes of administration represent systemic delivery of pharmacologically active substances, as vitamins enter the bloodstream from the site of administration and reach the target tissues or sites of action via systemic circulation.

### 4.4. Terminal Blood Collection and Serum Vitamin Measurements (Proof of Exposure)

At terminal anesthesia during euthanasia, blood was collected by capturing the blood jet after incision of the right atrium into a sterile tube. Samples were allowed to clot at room temperature for 20 min and then centrifuged for 10 min at 2000× *g*. Serum (supernatant) was carefully separated without disturbing the clot, aliquoted into labeled tubes, and stored at −80 °C until analysis. Terminal blood sampling was performed 7 days after the last vitamin B12 injection. For serum folate (nmol/L) and vitamin B12 (pmol/L) measurements, a random subset of three samples per experimental group was selected. Serum folate and vitamin B12 were quantified on an Access 2 immunoassay analyzer (Beckman Coulter, Brea, CA, USA, Immunoassay System) using chemiluminescent immunoassays (folate assay: REF A98032; vitamin B12/cobalamin assay: REF 33000). These measurements were performed as proof of systemic exposure to the administered vitamins (i.e., to confirm exposure), rather than to establish pharmacokinetic equivalence across regimens.

### 4.5. Gait Analysis

**Figure 6:**
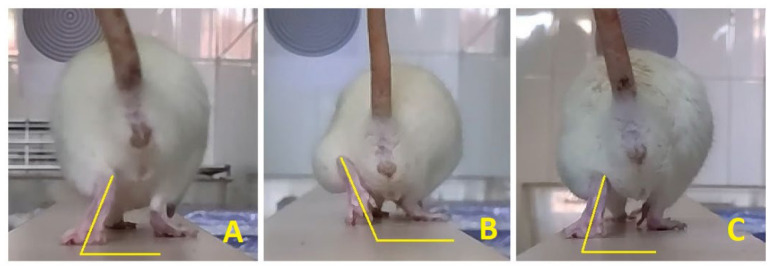
Foot-base angle measurements using single-frame motion analysis (SFMA). Single video frames from recordings of a rat during beam walking trials. Drawn lines show foot-base angles of the left foot, when the plantar surface of the foot was perpendicular to the beam surface. Panel (**A**) shows FBA before surgical lesion (D0), panel (**B**) shows FBA one day after injury (D1) and panel (**C**) shows FBA 8 weeks after injury (W8).

Recovery of the femoral nerve lesion was monitored by observing and recording gait on a wooden beam 10 cm wide and 2 m long (Figure 6). The wooden beam was wide enough so that the animals did not need to balance on it. The recording was performed according to the following schedule: 1 day before surgery (healthy control, D0), 1 and 7 days after surgery (D1, D7, respectively), and 2, 3, 4, 5, 6, 7 and 8 weeks after surgery (W2, 3, 4, 5, 6, 7, 8, respectively). For each animal, a rear-view gait recording at 120 fps was made. During each gait testing session, the animals were allowed to cross the plank several times toward the opposite end in order to obtain a sufficient number of frames for analysis. The only motivation for the animals to reach the other end of the plank was to access their home cage, which was placed on the opposite side. This type of testing did not require prior training of the animals, as rats naturally tend to seek the safety of their home cage. During the test, the rats were free to move along the plank and explore. The criteria for including frames in the analysis were as follows: frames were selected from video sequences in which the rat was walking straight ahead at an approximately constant speed. Sequences in which the rat was turning left or right, hopping, stopping, or running were not considered for the analysis. Frames were extracted at a specific phase of gait, when the plantar surface of the injured (left) foot was perpendicular to the walking surface, to measure the foot-base angle (FBA). The FBA of the rats was measured using ImageJ software (version 1.54, National Institutes of Health, Bethesda, MD, USA) on these frames. Statistical analysis of FBA values was performed to evaluate gait function recovery among the experimental groups and the control. For SFMA/FBA, video files were re-labeled with random identifiers by a team member not involved in scoring. FBA measurements were performed in ImageJ by an assessor blinded to treatment allocation and time point, with the file order randomly permuted.

### 4.6. Histological Analysis

Eight weeks after injury, animals were euthanized and peripheral nerve samples from the left femoral nerve were collected for histological preparation and microscopic analyses. Prepared femoral nerve sections were used to assess regeneration parameters, quantify axons in the motor branch, and evaluate myelination using the g-ratio. Histological analysis was performed as described previously [60]. Rats were deeply anesthetized via intraperitoneal ketamine/xylazine (ketamine 100 mg/kg; xylazine 8 mg/kg). After confirming anesthesia, the thoracic cavity was opened and transcardial perfusion was carried out as follows: 1 min with phosphate-buffered saline (PBS), then 10 min with 4% formaldehyde in 0.1 M sodium cacodylate buffer (pH 7.3). Left femoral nerves were removed for processing. Post-fixation employed 1% osmium tetroxide (Polysciences Europe, Eppelheim, Germany; product no. 20816-12-0) in 0.1 M sodium cacodylate (pH 7.3) for 1 h at room temperature, followed by graded ethanol dehydration and resin embedding. Semithin (1 μm) cross-sections or longitudinal sections were obtained from the quadriceps (“motor”) and saphenous (“sensory”) branches starting ~3 mm distal to the bifurcation. Sections were stained with 1% toluidine blue plus 1% borax (aqueous) for evaluation of axon numbers and myelination. Myelinated axons per cross-section were counted using a Neurolucida-controlled setup (MicroBrightField Europe, Magdeburg, Germany) on a Zeiss Axioskop microscope with a 100× oil-immersion objective (Carl Zeiss, Oberkochen, Germany). Morphometry recorded axon diameter and total fiber diameter (axon + myelin sheath) at matched locations within each section. Sampling used a square grid spaced at 60 μm projected in Neurolucida 2020.1.1, with randomized reference points. For myelinated profiles touching or crossing the vertical grid lines, the maximal diameter and the perpendicular diameter were measured, and the mean of the orthogonal pair was taken as the estimate. Myelination was summarized by the g-ratio, defined as the axon diameter to total fiber diameter; lower values indicate thicker myelin. All samples and image files carried coded identifiers; myelinated axon counts and g-ratio morphometry were performed by an assessor blinded to group identity. Codes were disclosed only after the database was locked.

### 4.7. Photographic Documentation

Micrographs were captured on a Zeiss Axiophot 2 microscope fitted with a digital camera module and operated via ZEN software, version 2.1 (Carl Zeiss, Oberkochen, Germany). Post-acquisition processing in Adobe Photoshop CS5 (Adobe Systems Inc., San Jose, CA, USA) comprised only unsharp masking, global brightness/contrast adjustment, and cropping; no other alterations were performed.

### 4.8. Statistical Analysis

Time-dependent changes in foot-base angle (FBA) were analyzed with a two-way re-peated-measures ANOVA including Time and Treatment factors, followed by Tukey’s post hoc tests. Repeated-measures models were applied to account for within-animal correlations across time points. Between-group comparisons of FBA at matched time points were additionally assessed with Tukey’s Honestly Significant Difference (HSD). Within-group progression between adjacent time points (weekly recovery) was examined using repeated-measures ANOVA combined with Holm-adjusted paired *t*-tests. Group differences in the numbers of myelinated axons were tested by one-way ANOVA with Tukey post hoc procedures. Distributions of g-ratio values in myelinated axons were compared using the Kolmogorov–Smirnov test. Data are presented as mean ± standard deviation (SD), and statistical significance was set at *p* < 0.05. Primary statistical analyses were conducted on coded group labels (A/B/C) to maintain blinding; group identities were revealed after finalization of the results and figures.

### 4.9. Literature Search (Rationale)

A systematic approach was employed to identify existing research, including cross-referencing of terms across the PubMed, Scopus, and Google Scholar databases. This targeted search—focusing on the comparative effects of folic acid and vitamin B12 specifically in the rat femoral-nerve model—yielded no prior head-to-head studies, which informed the study design.

## 5. Conclusions

This study demonstrates that high-dose vitamin B12 significantly improves both functional and structural recovery following femoral nerve injury in rats. Vitamin B12 treatment resulted in superior gait restoration and enhanced myelination compared to folic acid and control groups, indicating its strong neuroregenerative potential. Folic acid supplementation promoted an increase in the number of myelinated axons and contributed to early-phase recovery, but did not improve myelin thickness or late-phase functional outcomes. These findings highlight the importance of vitamin B12 in peripheral nerve repair and suggest that its use may offer a promising therapeutic approach for improving recovery after nerve injuries. Further research should explore optimal dosing strategies and long-term effects in clinical settings.

## Data Availability

Dataset available on request from the authors.

## Abbreviations

The following abbreviations are used in this manuscript:

Bax	BCL-2-associated X protein
BCL-2	B-cell lymphoma 2
BDNF	Brain-derived neurotrophic factor
CMAP	Compound muscle action potential
CON	Control
DNM3	Dynamin 3
Erk1/2	Extracellular signal-regulated kinase 1/2
FA	Folic acid
FBA	Foot-base angle
GPx	Glutathione peroxidase
MBP	Myelin basic protein
NGF	Nerve growth factor
NMJ	Neuromuscular junction
PNI	Peripheral nerve injury
s.c.	Subcutaneous
SFMA	Single-frame motion analysis
SOD	Superoxide dismutase
WD	Wallerian degeneration
